# Opioid-Free and Opioid-Sparing Pain Control in Surgical Anesthesia: A Scoping Review of Multimodal Analgesia, Regional Techniques, and Recovery Outcomes

**DOI:** 10.7759/cureus.113435

**Published:** 2026-07-27

**Authors:** Prakriti Shukla, Ananya Sharma, Sanjit Prasad, Rachana Singh, Alpeshkumar J Parmar, Rajesh Kumar

**Affiliations:** 1 Department of Anaesthesiology and Critical Care, Dr. Ram Manohar Lohia Institute of Medical Sciences, Lucknow, IND; 2 Department of Anaesthesiology, University of Health Science, Rohtak, IND; 3 Department of General Surgery, All India Institute of Medical Sciences, Patna, Patna, IND; 4 Department of General Anaesthesia, University College of Medical Sciences and Guru Teg Bahadur Hospital, New Delhi, IND; 5 Department of General Surgery, Dr. N. D. Desai Faculty of Medical Science and Research, Dharmsinh Desai University, Nadiad, IND; 6 Department of Anaesthesiology, All India Institute of Medical Sciences, Patna, Patna, IND

**Keywords:** enhanced recovery after surgery, multimodal analgesia, opioid-free anaesthesia, opioid-sparing analgesia, regional anaesthesia

## Abstract

Surgical anesthesia has historically relied on opioids to control intraoperative and postoperative pain, but opioid-related adverse effects such as respiratory depression, sedation, ileus, postoperative nausea and vomiting, delayed mobilization, tolerance, hyperalgesia, and prolonged opioid use have encouraged a shift toward safer perioperative analgesic models. In this scoping review, opioid-free anesthesia (OFA) is defined as the complete avoidance of opioid administration during the intraoperative period, whereas opioid-sparing anesthesia (OSA) is defined as an anesthetic approach that intentionally reduces, but does not eliminate, intraoperative opioid use through non-opioid pharmacologic agents, regional anesthesia, local anesthetic techniques, or multimodal analgesic strategies. Despite growing interest in opioid-free and opioid-sparing anesthesia, available evidence remains inconsistent because studies differ in drug combinations, dosing schedules, surgical populations, regional techniques, rescue analgesia protocols, and recovery measurements. This scoping review evaluates contemporary opioid-free pain control in surgical anesthesia, focusing on multimodal pharmacology, regional anesthesia, fascial plane blocks, enhanced recovery pathways, patient-centered outcomes, safety, and risk stratification. Literature published between January 2018 and May 2026 was searched in PubMed/MEDLINE, Scopus, Web of Science, and Google Scholar. Forty-six articles were included and narratively mapped across pharmacologic strategies, regional techniques, enhanced recovery after surgery (ERAS) pathways, recovery outcomes, safety considerations, and risk-stratification themes. Current evidence suggests that opioid-free and opioid-sparing strategies may reduce perioperative opioid exposure and selected opioid-related adverse effects in appropriately selected procedures and patients, although findings remain heterogeneous. The strongest benefit appears when non-opioid drugs, regional techniques, and ERAS principles are combined within procedure-specific pathways. Opioid-free anesthesia should be considered a flexible, individualized strategy rather than a universal replacement for balanced anesthesia.

## Introduction and background

Opioids have traditionally been used for intraoperative analgesia, hemodynamic control, and postoperative pain relief. Their use is associated with respiratory depression, sedation, postoperative nausea and vomiting, ileus, delayed mobilization, tolerance, opioid-induced hyperalgesia, and persistent postoperative opioid use [1,2]. These concerns have encouraged recovery-focused perioperative strategies that aim to maintain analgesia while reducing opioid exposure and opioid-related complications [3,4].

Opioid-free anesthesia (OFA) refers to the planned avoidance of intraoperative opioids, whereas opioid-sparing anesthesia (OSA) reduces but does not eliminate opioid use, retaining opioids for limited administration or rescue when clinically required [4,5]. Both approaches rely on multimodal analgesia using systemic non-opioid agents, regional anesthesia, local infiltration, peripheral nerve blocks, or fascial plane blocks. Common agents include acetaminophen, nonsteroidal anti-inflammatory drugs, cyclooxygenase-2 inhibitors, ketamine, dexmedetomidine, clonidine, intravenous lidocaine, magnesium sulfate, and selected gabapentinoids [2,6]. Regimen selection should be individualized according to the surgical procedure, expected pain severity, comorbidities, opioid tolerance, contraindications, and recovery goals [6,7].

The clinical effects of OFA and OSA vary across surgical settings and patient populations. These strategies may be particularly relevant in patients with obesity, obstructive sleep apnea, respiratory vulnerability, chronic opioid exposure, or increased risk of prolonged postoperative opioid use [5,6]. Evidence suggests that opioid minimization may reduce opioid consumption and selected adverse effects, including postoperative nausea and vomiting, but complete opioid avoidance does not consistently improve recovery when both comparison groups receive effective multimodal analgesia [8-10]. OFA and OSA should therefore be considered flexible components of enhanced recovery after surgery (ERAS) pathways rather than universal replacements for balanced anesthesia. Their success should be assessed through adequate pain control, adverse events, functional recovery, patient-centered outcomes, and access to timely rescue analgesia [4,11].

Current evidence remains heterogeneous because studies differ in surgical populations, drug combinations, regional techniques, dosing schedules, rescue analgesia protocols, and outcome measures [11,12]. A structured mapping of the available evidence is needed to clarify the clinical applications, safety considerations, recovery outcomes, and remaining knowledge gaps related to opioid-free and opioid-sparing perioperative care.

Objective of the review

This scoping review aims to map evidence published from January 2018 to May 2026 on OFA and OSA across surgical specialties. It evaluates systemic non-opioid agents, regional anesthesia, fascial plane blocks, and enhanced recovery pathways in relation to perioperative opioid use, postoperative pain, opioid-related adverse events, functional recovery, patient safety, and patient-centered outcomes. It also identifies procedure-specific applications, patient selection considerations, safety concerns, and evidence gaps without generating pooled effect estimates.

Methodology

Review Design

This article was designed as a scoping review because the evidence on OFA and OSA is broad, clinically heterogeneous, and distributed across multiple surgical specialties, pharmacologic regimens, regional anesthesia techniques, ERAS pathways, and recovery outcomes. A scoping review approach was selected to map the range of available evidence, identify major clinical themes, summarize current practice patterns, and highlight knowledge gaps rather than to answer a narrowly defined intervention-comparator question or generate pooled effect estimates. Therefore, no statistical pooling, meta-regression, formal risk-of-bias scoring, certainty-of-evidence grading, P-values, or confidence intervals were calculated. For this review, OFA was operationally defined as complete intraoperative opioid avoidance, while OSA was operationally defined as intentional intraoperative opioid reduction without complete opioid elimination.

Literature Search

This scoping review used a structured literature search to identify recent evidence on opioid-free and opioid-sparing pain control in surgical anesthesia. The final literature search was conducted on May 20, 2026. Literature published from January 2018 to May 2026 was searched in PubMed/MEDLINE, Scopus, Web of Science, and Google Scholar. The search terms included “opioid-free anesthesia,” “opioid-sparing anesthesia,” “multimodal analgesia,” “non-opioid perioperative analgesics,” “regional anesthesia,” “fascial plane blocks,” “enhanced recovery after surgery,” “ERAS,” “postoperative pain,” “perioperative outcomes,” and “postoperative recovery.” Boolean operators (“AND,” “OR,” and “NOT”) were used to combine terms. The main search combination was: (“opioid-free anesthesia” OR “opioid-sparing anesthesia”) AND (“multimodal analgesia” OR “regional anesthesia” OR “fascial plane block” OR “enhanced recovery after surgery” OR “ERAS” OR “postoperative recovery”). Reference lists of relevant review articles and included studies were also screened manually to identify additional clinically relevant articles.

Eligibility Criteria

Articles were included if they addressed perioperative OFA, OSA, systemic non-opioid analgesic strategies, regional anesthesia, fascial plane blocks, enhanced recovery pathways, patient-centered outcomes, postoperative opioid exposure, safety, risk stratification, or surgical recovery outcomes. Eligible evidence included original human clinical studies, such as randomized controlled trials, cohort studies, comparative observational studies, and case series, together with systematic reviews, meta-analyses, clinical guidelines, consensus statements, and clinically relevant narrative reviews. Original clinical studies were specifically examined to provide direct evidence on the effectiveness, safety, and recovery outcomes of OFA and OSA, whereas review articles and guidance documents were used to map the broader evidence base, clinical context, and implementation considerations. Articles were excluded if they were published before 2018, were not written in English, were animal or laboratory-only studies, were unrelated to surgical perioperative pain management, lacked clinically relevant outcome data, focused only on chronic non-surgical pain, or did not address opioid-free or opioid-sparing perioperative care.

Study Identification and Screening

**Figure 1:**
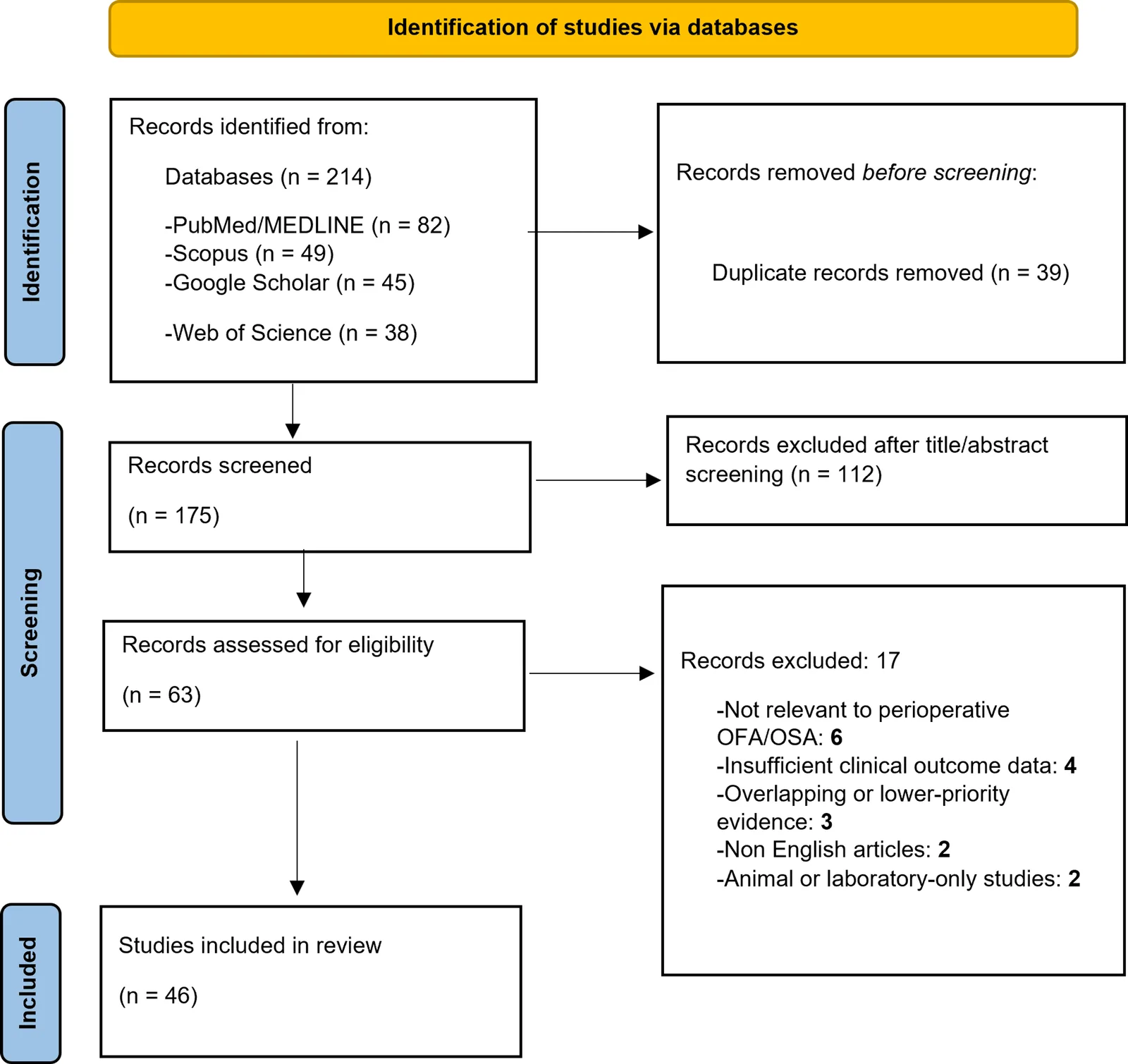
Preferred Reporting Items for Systematic Reviews and Meta-Analyses (PRISMA)-style flow diagram of study identification and selection

A total of 214 records were identified through PubMed/MEDLINE, Scopus, Web of Science, and Google Scholar. After removal of 39 duplicates, 175 records underwent title and abstract screening, of which 112 were excluded as unrelated to perioperative OFA, OSA, or surgical pain management. Sixty-three full-text articles were assessed against the predefined eligibility criteria. Seventeen articles were excluded because they were not sufficiently relevant to perioperative OFA or OSA (n=6), lacked clinically relevant outcome data (n=4), duplicated evidence or provided no additional extractable clinical information (n=3), were not published in English (n=2), or involved animal or laboratory-only research (n=2). Application of the predefined date, language, population, topic, and outcome criteria resulted in the inclusion of 46 articles. Individual human clinical studies were prioritized when describing the direct effectiveness, safety, and recovery outcomes of OFA and OSA, whereas systematic reviews, meta-analyses, guidelines, and narrative reviews were used to map the broader evidence base and provide clinical context. The study-selection process is presented in the Preferred Reporting Items for Systematic Reviews and Meta-Analyses (PRISMA)-style flow diagram in Figure 1.

Study Selection Process

Studies were selected by two reviewers independently. The reviewers screened titles and abstracts first, followed by full-text assessment of potentially eligible articles. Disagreements were resolved through discussion with a third reviewer. Articles were included if they addressed perioperative OFA or OSA, systemic non-opioid analgesics, regional anesthesia, fascial plane blocks, enhanced recovery pathways, patient-centered outcomes, safety, risk stratification, or postoperative recovery. Articles published before 2018, non-English articles, animal studies, laboratory-only studies, non-surgical studies, and articles unrelated to perioperative pain management were excluded. When multiple articles addressed the same clinical question, recent primary human studies reporting direct perioperative outcomes were prioritized, including randomized controlled trials, comparative cohort studies, prospective clinical studies, and qualitative clinical investigations. Review articles and meta-analyses were retained mainly for background, mechanistic interpretation, contextualization of findings, and identification of broader evidence gaps.

Data Charting

Data were charted from eligible articles according to predefined domains. These domains included article type, surgical setting, anesthetic or analgesic intervention, comparator approach where applicable, regional technique, postoperative opioid exposure, pain outcomes, postoperative nausea and vomiting, sedation, respiratory outcomes, bowel recovery, mobilization, length of stay, discharge readiness, patient-centered outcomes, safety concerns, risk-stratification considerations, and relevance to ERAS pathways.

Evidence Mapping and Synthesis

Evidence was mapped across major clinical themes, including surgical pain mechanisms, systemic non-opioid pharmacologic strategies, regional anesthesia and fascial plane blocks, ERAS-based pathways, patient-centered recovery outcomes, safety, risk stratification, patient selection, limitations, and future directions. Because this was a scoping review, findings were synthesized narratively rather than quantitatively. Primary human clinical studies, including randomized controlled trials, cohort studies, comparative observational studies, qualitative studies, and case series, were given greater interpretive weight when evaluating the direct effectiveness, safety, and recovery outcomes of OFA and OSA. Systematic reviews, meta-analyses, clinical guidelines, consensus statements, and narrative reviews were used mainly to define concepts, describe mechanisms, provide broader clinical context, and identify implementation considerations and evidence gaps. Conclusions regarding clinical effectiveness were not based solely on review-level evidence.

## Review

Pathophysiology of surgical pain and targets for non-opioid analgesia

**Figure 2:**
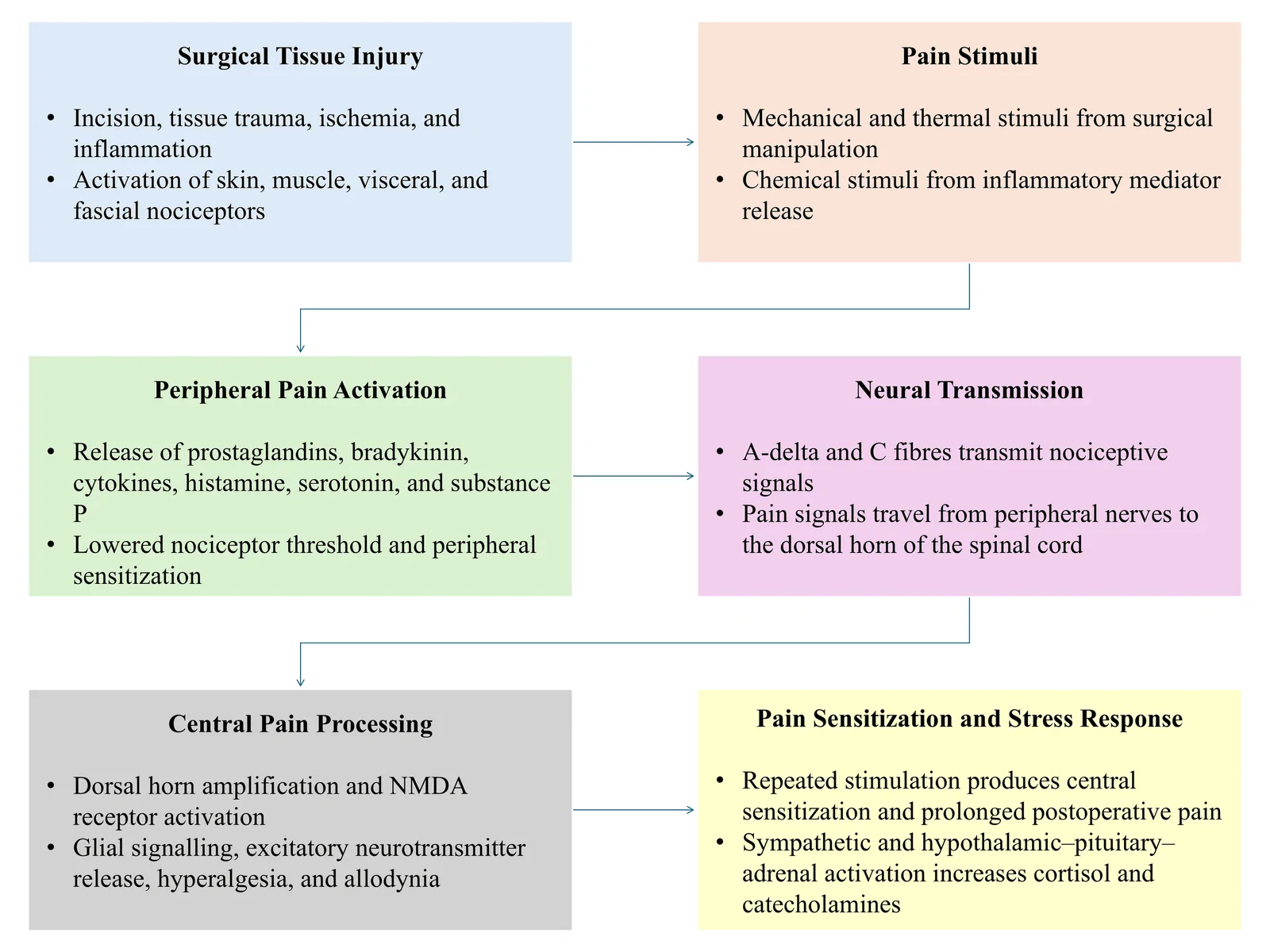
Pathophysiology of surgical pain and targets for non-opioid analgesia Original figure created by the authors using Microsoft PowerPoint

Surgical pain arises from tissue injury, inflammatory mediator release, peripheral nociceptor activation, neural transmission, central sensitization, and activation of the neuroendocrine stress response [13-15]. These mechanisms provide the rationale for multimodal analgesia, in which systemic non-opioid agents and regional techniques target complementary inflammatory, nociceptive, sensitization, and autonomic pathways [16-18]. Regional anesthesia and fascial plane blocks further reduce nociceptive transmission at peripheral, fascial, plexus-based, or neuraxial sites. These approaches may reduce opioid exposure, but adequate analgesia and timely rescue opioid administration should remain available when clinically required. Figure 2 summarizes the principal pain pathways and corresponding non-opioid targets.

Systemic non-opioid pharmacological strategies

Systemic non-opioid pharmacologic strategies provide an important foundation for OFA and OSA. Their role follows from the pathophysiology of surgical pain, which involves inflammation, nociceptive transmission, central sensitization, autonomic responses, and procedure-specific pain mechanisms. These agents target several of these pathways and may reduce, but not always eliminate, perioperative opioid requirements [19,20]. They are typically used within multimodal analgesia, in which drugs with different mechanisms are combined to address nociceptive, inflammatory, neuropathic, and central sensitization components of surgical pain [21].

Acetaminophen, nonsteroidal anti-inflammatory drugs, and cyclooxygenase-2 inhibitors are commonly incorporated into multimodal analgesic regimens to provide central and anti-inflammatory analgesia, with use individualized according to renal, gastrointestinal, cardiovascular, and bleeding risks [19]. Ketamine may reduce central sensitization, opioid tolerance, and opioid-induced hyperalgesia, particularly in painful procedures and patients with prior opioid exposure [19,20]. Alpha-2 adrenergic agonists, intravenous lidocaine, magnesium sulfate, and selected gabapentinoids may also be incorporated according to the expected pain mechanism, surgical context, and patient risk, with individualized dosing and monitoring for sedation, hemodynamic effects, systemic toxicity, renal impairment, and delayed recovery [19].

The clinical value of systemic non-opioid pharmacology depends on procedure-specific application rather than routine replacement of opioids with a fixed drug bundle. Newer multimodal approaches are increasingly used in total joint arthroplasty, where they may support early mobilization, rehabilitation, and reduced opioid exposure [21]. In cancer surgery, analgesic planning should account for nociceptive, inflammatory, neuropathic, and disease-related pain mechanisms, supporting individualized pharmacologic and non-pharmacologic interventions [22]. In patients with opioid use disorder or prolonged opioid exposure, non-opioid approaches may support opioid reduction, although treatment should remain individualized and should not compromise adequate analgesia [23]. Regional analgesia provides an additional opioid-sparing strategy by interrupting nociceptive transmission at peripheral, plexus-based, fascial, or neuraxial targets and is particularly relevant to cardiothoracic surgical pathways [24].

Overall, systemic non-opioid analgesics provide a mechanistically rational approach to perioperative pain management when selected according to surgical procedure, patient comorbidities, contraindications, expected pain severity, and recovery goals. Their value is greatest when integrated with regional anesthesia, fascial plane blocks, enhanced recovery pathways, and clearly defined rescue analgesia plans. These strategies should reduce unnecessary opioid exposure without implying that opioids are obsolete; opioids may remain necessary for rescue analgesia, severe pain, or hemodynamic control in selected patients. The included evidence base was re-examined to prioritize individual human clinical studies wherever they directly evaluated opioid-free or opioid-sparing interventions. Review-level sources were retained only for drug-specific mechanisms, clinical indications, and safety considerations when corresponding individual studies were not available within the included literature. Table 1 presents the major systemic non-opioid pharmacologic strategies, specific drugs and methods used, clinical roles, and supporting evidence in opioid-free and opioid-sparing surgical anesthesia.

**Table 1:** Systemic non-opioid pharmacologic strategies, methods, and clinical considerations NSAIDs: nonsteroidal anti-inflammatory drugs; COX-2: cyclooxygenase-2; NMDA: N-methyl-D-aspartate.

Non-opioid strategy	Specific drug or method used	Main perioperative role	Evidence and clinical context	Key consideration	References
Multimodal systemic analgesia	Combined use of acetaminophen or paracetamol, NSAIDs or COX-2 inhibitors, ketamine, alpha-2 adrenergic agonists, intravenous lidocaine, magnesium sulfate, and selected gabapentinoids	Targets multiple inflammatory, nociceptive, central sensitization, and autonomic pathways	A comparative cohort study in video-assisted thoracoscopic surgery evaluated opioid-free and opioid-sparing multimodal protocols [10], while broader evidence has summarized multimodal opioid-free approaches across surgical settings [2].	The regimen should be individualized because no single standardized combination is suitable for all procedures or patients.	[2,10]
Acetaminophen or paracetamol	Scheduled oral or intravenous administration within a multimodal regimen	Foundational component of perioperative multimodal analgesia	Perioperative literature describes acetaminophen or paracetamol as a commonly used central analgesic adjunct.	Total daily dosing should be adjusted according to hepatic function and concurrent acetaminophen-containing medications.	[16,17]
NSAIDs and COX-2 inhibitors	Oral or intravenous administration for inflammatory pain control	Reduces prostaglandin-mediated inflammation and peripheral sensitization	Non-opioid perioperative analgesic literature supports their inclusion in opioid-sparing regimens when clinically appropriate.	Renal function, gastrointestinal risk, bleeding risk, and cardiovascular comorbidity should be assessed before use.	[17,19]
Ketamine	Low-dose intravenous bolus or infusion as a multimodal adjunct	Targets central sensitization and may benefit patients with severe pain or opioid tolerance	Ketamine acts as an NMDA receptor antagonist and may reduce opioid tolerance, hyperalgesia, and perioperative opioid requirements.	Monitoring is required for psychotomimetic, cardiovascular, and recovery-related adverse effects.	[17,19]
Alpha-2 adrenergic agonists	Dexmedetomidine or clonidine administered as an intravenous or protocol-based adjunct	Provides analgesic, sedative, anxiolytic, and sympatholytic effects	Alpha-2 agonists are used as opioid-sparing adjuncts within multimodal anesthesia protocols.	Monitoring is required for bradycardia, hypotension, excessive sedation, and delayed recovery.	[16,19]
Intravenous lidocaine	Perioperative intravenous infusion	Provides analgesic, anti-inflammatory, and anti-hyperalgesic effects, particularly for abdominal or visceral pain	Intravenous lidocaine is described as a systemic opioid-sparing adjunct in perioperative analgesic practice.	Careful dose control and monitoring are required because excessive plasma concentrations may cause neurologic or cardiac toxicity.	[17,19]
Other selected adjuncts	Magnesium sulfate and selected gabapentinoids incorporated selectively into multimodal protocols	Provides complementary analgesic and neuromodulatory effects	These agents may be used as adjuncts according to the expected pain mechanism and individual patient risk.	Selection should consider renal function, sedation risk, respiratory vulnerability, and institutional protocols.	[16,19]

Regional anesthesia and fascial plane blocks in opioid-free surgery

Regional anesthesia provides the anatomic component of opioid-free and opioid-sparing perioperative analgesia. Its role follows from the limitations of systemic non-opioid agents, which may be insufficient for procedures with substantial somatic, visceral, or thoracic pain. By interrupting nociceptive transmission before central amplification occurs, regional techniques can strengthen multimodal analgesia and support recovery-focused perioperative care. Regional anesthesia reduces nociceptive transmission at peripheral, fascial, neuraxial, or plexus-based targets before pain signals reach the central nervous system [24]. These techniques can reduce perioperative opioid requirements, limit opioid-related adverse events, and support recovery outcomes such as early mobilization, respiratory stability, and patient comfort [25,26]. Regional analgesia is particularly relevant in thoracic and selected cardiac procedures because postoperative pain can impair coughing, deep breathing, pulmonary mechanics, and mobilization [24]. Thoracic epidural and paravertebral techniques provide effective analgesia, but their use may be limited by anticoagulation, hemodynamic instability, technical complexity, and procedure-related risks [26]. In selected cardiac surgery patients, fascial plane blocks may be considered as part of multimodal analgesia, especially when neuraxial techniques are unsuitable or contraindicated [26]. Regional anesthesia can support opioid minimization by targeting procedure-specific pain pathways, but it should not be treated as a universal replacement for systemic analgesia or rescue opioids. The literature supporting regional techniques was re-examined across cardiothoracic and spinal surgical settings. Individual clinical evidence was prioritized where available, including the randomized controlled trial of continuous pecto-intercostal fascial plane block after open cardiac surgery [27]. Focused regional-anesthesia literature was retained to describe technique selection, anatomical coverage, safety considerations, and procedure-specific implementation [26,28].

Fascial plane blocks provide targeted local anesthetic spread within defined fascial compartments [25]. Ultrasound guidance improves anatomic visualization, needle placement, and local anesthetic deposition, which may reduce complications compared with deeper neuraxial approaches [25]. Techniques relevant to thoracic and cardiac surgery include erector spinae plane block, serratus anterior plane block, pecto-intercostal fascial plane block, transversus thoracic muscle plane block, and pectoral nerve blocks [26,27]. Other fascial plane blocks, including transversus abdominis plane block, quadratus lumborum block, and rectus sheath block, are more relevant to abdominal wall and abdominal surgical procedures [26,27]. The pecto-intercostal fascial plane block has shown analgesic efficacy after median sternotomy in open cardiac surgery [27]. Continuous catheter-based local anesthetic infusion may extend analgesia when a single-shot block provides insufficient duration or when sustained opioid-sparing analgesia is required [27]. These examples show that fascial plane blocks are not interchangeable; their value depends on the surgical field, expected pain distribution, block duration, and local expertise.

Spinal surgery is another setting in which fascial plane blocks, particularly the erector spinae plane block, have been studied because postoperative pain may arise from paraspinal muscle trauma, bony manipulation, and extensive soft-tissue injury [28]. Recent literature suggests that fascial plane blocks may reduce postoperative pain scores, opioid consumption, and early recovery limitations in selected spinal procedures. However, the optimal technique, local anesthetic volume, concentration, and timing remain under investigation [28]. This supports procedure-specific rather than universal application of regional techniques in opioid-free and opioid-sparing pathways.

Regional anesthesia and fascial plane blocks are not risk-free. Potential complications include block failure, local anesthetic systemic toxicity, vascular puncture, infection, hematoma, nerve injury, pneumothorax, rebound pain after block resolution, and delayed recognition of surgical complications [25]. Safe implementation requires ultrasound expertise, appropriate dosing, preparedness for toxicity management, postoperative monitoring, and clearly defined rescue analgesia plans [25,26]. These limitations are important because inadequate or failed regional analgesia may require timely opioid rescue to prevent undertreated pain and delayed recovery.

Overall, available evidence supports regional anesthesia as a procedure-specific component of opioid-free and opioid-sparing care rather than a uniform intervention. Regional anesthesia and fascial plane blocks are applicable across multiple surgical settings, including thoracic, cardiac, abdominal, and spinal procedures; the cardiac and spinal examples discussed in this section represent selected applications rather than the full clinical scope of these techniques. Their effectiveness depends on the surgical site, expected pain distribution, block technique and duration, patient selection, local anesthetic dosing, operator expertise, monitoring, and integration with systemic non-opioid analgesia and rescue analgesia pathways. Table 2 summarizes the representative applications of regional anesthesia and fascial plane blocks across thoracic, cardiac, abdominal, and spinal surgery, together with their principal analgesic roles, limitations, and safety considerations.

**Table 2:** Procedure-specific applications of regional anesthesia and fascial plane blocks

Technique or surgical application	Main role	Representative evidence or clinical context	Key point	References
Regional and neuraxial anesthesia	Interrupts nociceptive transmission at peripheral, plexus-based, or neuraxial targets	Regional analgesic techniques are used across thoracic, cardiac, abdominal, orthopedic, and other major surgical procedures as components of multimodal analgesia.	Technique selection should be based on the operative site, expected pain distribution, contraindications, and available expertise.	[24,25]
Thoracic and cardiac surgery	Controls chest-wall, thoracic, and sternotomy pain	Thoracic epidural, paravertebral, erector spinae plane, serratus anterior plane, pectoral, and parasternal fascial plane techniques may be incorporated into procedure-specific pathways.	Effective analgesia may support coughing, deep breathing, mobilization, and respiratory stability.	[24,25]
Continuous pecto-intercostal fascial plane block	Provides prolonged analgesia after median sternotomy	Zhang et al. evaluated continuous pecto-intercostal fascial plane block in a randomized controlled trial of patients undergoing open cardiac surgery.	Continuous local anesthetic administration may extend analgesia beyond a single-shot block.	[27]
Abdominal and abdominal-wall surgery	Targets somatic pain arising from the abdominal wall	Transversus abdominis plane, quadratus lumborum, and rectus sheath blocks are selected according to incision location and expected anatomical coverage.	These blocks may complement systemic analgesia but may not provide complete control of visceral pain.	[26]
Spinal surgery	Targets paraspinal and posterior spinal pain	Erector spinae plane and related fascial plane techniques have been investigated for postoperative analgesia after spinal procedures.	They may reduce early pain and opioid requirements, although optimal technique, dose, and timing remain uncertain.	[28]
Other procedure-specific applications	Provides localized analgesia according to the operative field	Peripheral and fascial plane techniques may be selected in other surgical settings when their anatomical coverage corresponds to the incision and anticipated pain distribution.	Blocks are not interchangeable and should be selected according to surgical anatomy, patient risk, and operator expertise.	[25,26]
Safety and implementation	Reduces block-related harm	Regional techniques require appropriate patient selection, ultrasound guidance, local anesthetic dose control, monitoring, and a rescue analgesia plan.	Potential concerns include block failure, local anesthetic systemic toxicity, hematoma, pneumothorax, rebound pain, and delayed rescue analgesia.	[25,26]

Enhanced recovery after surgery and opioid-free pathways

Enhanced recovery after surgery (ERAS) provides a structured framework for integrating systemic non-opioid agents, regional techniques, patient education, early mobilization, bowel recovery, oral intake, and discharge planning [29,30]. Within this framework, OFA and OSA should support functional recovery and reduce opioid-related adverse effects such as sedation, postoperative nausea and vomiting, ileus, urinary retention, and respiratory depression [30,31]. Opioid reduction should not be treated as an isolated endpoint; adequate analgesia, recovery quality, and timely rescue medication must remain central to perioperative care. ERAS-based multimodal regimens may combine systemic non-opioid agents, local infiltration, neuraxial techniques, peripheral nerve blocks, and fascial plane blocks [30,32]. Selection should reflect the surgical procedure, anticipated pain severity, comorbidities, contraindications, and need for rescue opioids. In gastrointestinal surgery, opioid minimization may support bowel motility, reduce nausea and ileus, and facilitate earlier feeding and ambulation [30,33]. Patient counseling is also important because expectations, communication, perceived safety, nausea control, and confidence in recovery influence acceptance of opioid-free pathways [33].

Clinical evidence indicates that the effects of OFA and OSA depend on the quality of the multimodal regimen, surgical procedure, regional technique, patient characteristics, and selected recovery outcomes. In video-assisted thoracoscopic surgery, complete opioid avoidance did not improve postoperative recovery compared with opioid-sparing anesthesia when both groups received multimodal analgesia [10]. A randomized trial in open cardiac surgery supported continuous pecto-intercostal fascial plane block as a procedure-specific opioid-sparing technique [27]. Qualitative evidence from bariatric surgery also showed that communication, expectations, nausea control, and confidence in recovery shape the patient experience of opioid-free care [33]. Procedure-specific perioperative planning remains important in colorectal and oncologic surgery, where surgical specialization, disease extent, operative approach, and multidisciplinary coordination influence postoperative care and recovery pathways [34,35]. Evidence-based gynecologic surgical programs similarly emphasize standardized perioperative practices, patient safety, and structured recovery planning [36]. A spine-surgery case series described a combined non-opioid regimen for OFA and reported satisfactory perioperative analgesia, hemodynamic stability, patient satisfaction, and postoperative recovery; these findings should be interpreted cautiously because the study lacked a comparative control group [37]. These sources provide procedure-specific clinical context but do not establish the comparative effectiveness of OFA or OSA. Table 3 summarizes the principal ERAS components, recovery goals, analgesic approaches, patient-counseling requirements, and implementation considerations.

**Table 3:** Enhanced Recovery After Surgery (ERAS) and opioid-free pathways ERAS: enhanced recovery after surgery; OFA: opioid-free anesthesia; OSA: opioid-sparing anesthesia

ERAS component	Main role	Key point	References
Enhanced Recovery After Surgery pathway	Coordinates perioperative recovery	Supports mobilization, bowel recovery, oral intake, and discharge readiness.	[31,33]
Opioid-free or opioid-sparing analgesia	Reduces opioid-related recovery delays	May reduce nausea, ileus, sedation, and respiratory depression when adequate analgesia is maintained.	[30,31]
Multimodal analgesia	Combines non-opioid drugs and regional techniques	Maintains analgesia while reducing perioperative opioid exposure.	[32,33]
Opioid minimization in gastrointestinal surgery	Supports bowel recovery within ERAS pathways	Reducing perioperative opioid exposure may lessen opioid-related ileus, nausea, and delayed gastrointestinal motility, thereby facilitating earlier oral intake and mobilization.	[30,31]
Preoperative counseling for OFA/OSA pathways	Prepares patients for the analgesic plan and recovery expectations	Explaining expected pain, non-opioid methods, rescue opioid availability, nausea control, and mobilization goals may improve acceptance, reduce anxiety, and support adherence to opioid-free or opioid-sparing recovery pathways.	[29,33]
Implementation challenges	Guides safe adoption	Protocols require adaptation according to the procedure, patient risk, institutional resources, and availability of rescue analgesia.	[30,33]

Patient-centered outcomes and recovery metrics

Patient-centered evaluation of OFA and OSA should include both analgesic efficacy and postoperative recovery [38]. Relevant measures include postoperative pain at rest and during movement, cumulative opioid consumption, rescue analgesia requirements, postoperative nausea and vomiting, sedation, respiratory events, mobilization, discharge readiness, patient satisfaction, and return to usual function [38,39].

**Figure 3:**
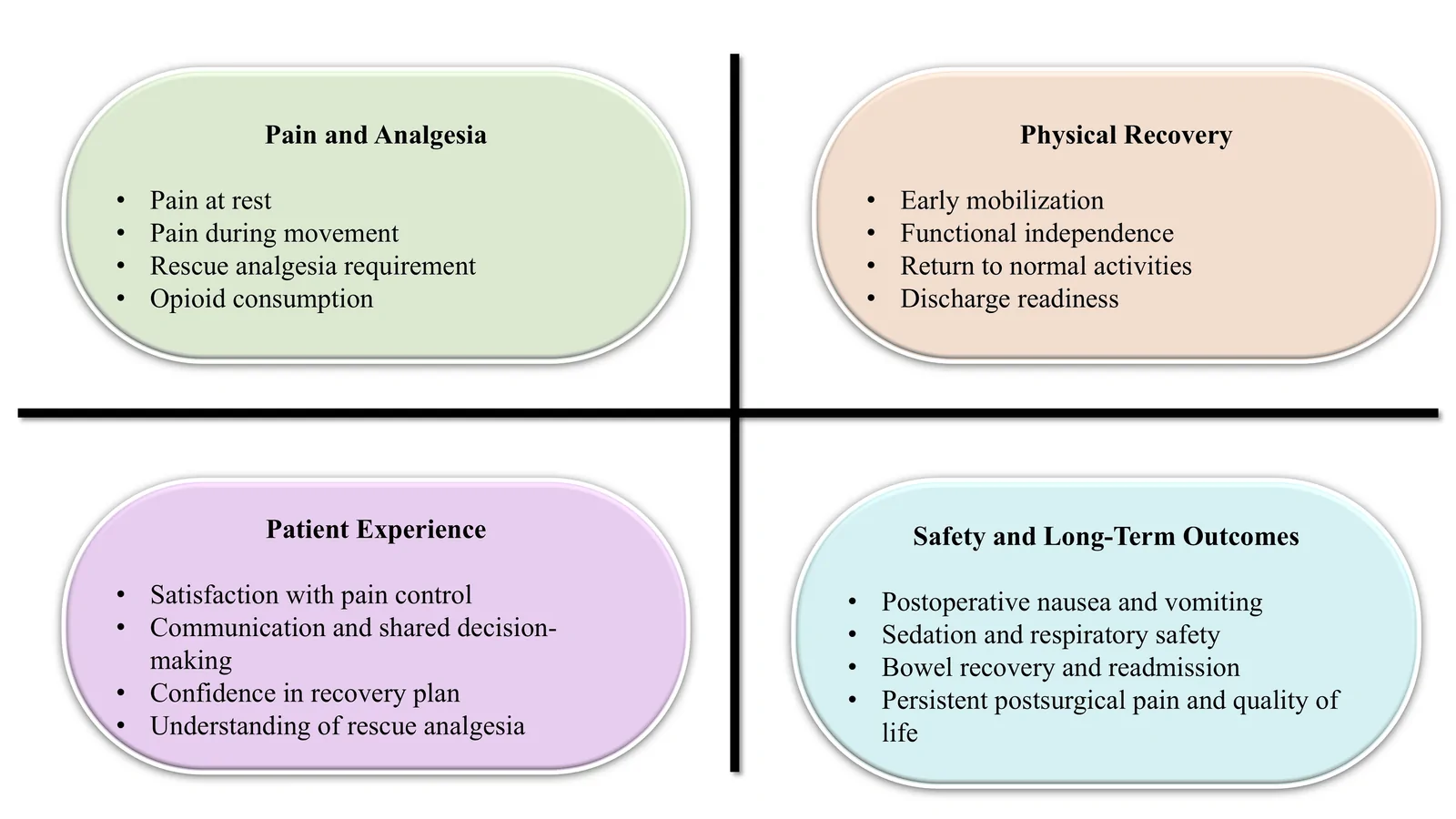
Patient-centered outcomes and recovery metrics in opioid-free and opioid-sparing anesthesia Original figure created by the authors using Microsoft PowerPoint

Recovery quality may also be evaluated using validated multidimensional instruments, such as Quality of Recovery questionnaires, which assess domains including pain, physical comfort, emotional state, independence, and psychological support [38,40]. Additional short-term outcomes include bowel recovery, oral intake, length of stay, and readmission [39,40]. Outcome assessment should extend beyond the immediate postoperative period because early reductions in pain or opioid consumption may not reflect sustained functional recovery. Longitudinal evaluation should consider persistent postsurgical pain, continued opioid use, functional independence, and health-related quality of life [40,41]. OFA and OSA should therefore be considered effective only when reduced opioid exposure is accompanied by adequate pain control, acceptable recovery-quality scores, fewer adverse events, and preserved functional recovery. Figure 3 presents the key patient-centered outcomes and recovery metrics used to assess OFA and OSA.

Safety, risk stratification, and patient selection

Safety, risk stratification, and patient selection are essential to the responsible implementation of OFA and OSA. Opioid minimization can be considered successful only when it preserves effective analgesia, avoids preventable adverse events, and supports equitable recovery. The benefits of opioid reduction must be weighed against the risks of insufficient analgesia, adverse drug reactions, hemodynamic instability, delayed rescue analgesia, and unequal access to appropriate pain control [42]. Surgical patients differ in pain sensitivity, comorbidity burden, psychological profile, prior opioid exposure, substance-use history, procedure type, social support, and recovery expectations; therefore, a uniform opioid-free protocol is clinically inappropriate [42].

Risk assessment is important for identifying patients at risk of sustained prescription opioid use after surgery [42]. A validated bedside risk assessment model for sustained postoperative opioid use highlights the importance of preoperative variables in perioperative planning. These tools can help identify patients who may benefit from opioid-minimizing strategies, closer follow-up, and structured discharge prescribing [42]. Relevant approaches include the Outpatient Arthroplasty Risk Assessment score and validated bedside prediction models for sustained postoperative opioid use or opioid overdose [43-45]. These tools should support, rather than replace, individualized clinical judgment. Risk stratification should also account for communication, patient expectations, perceived pain severity, clinician assumptions, and disparities in care [43]. Evidence on opioid prescribing disparities indicates that risk assessment should not be applied in isolation or in ways that cause undertreatment of pain [43]. Patient-centered discussions should address expected postoperative pain, non-opioid options, rescue analgesia, functional recovery goals, and concerns about opioid dependence or undertreatment [43]. This approach helps ensure that opioid minimization remains a safety-focused and equitable strategy rather than a rigid restriction on analgesic access.

Procedure-specific selection is also required. In outpatient joint arthroplasty, risk assessment scores are used to identify patients suitable for accelerated recovery and same-day discharge pathways [44]. These models show that recovery with reduced opioid exposure depends not only on the analgesic regimen but also on patient fitness, medical stability, home support, baseline mobility, and ability to manage pain after discharge [44]. More flexible analgesic planning may be required in patients with complex comorbidities, frailty, severe baseline pain, chronic opioid use, limited mobility, or inadequate social support [44]. Patient selection should therefore integrate surgical risk, functional status, home environment, and post-discharge safety rather than focusing only on intraoperative opioid avoidance.

Additional safeguards are needed for patients receiving chronic opioid therapy or those at risk of opioid misuse. In patients prescribed chronic opioid therapy, opioid-overdose prediction models indicate that clinical, behavioral, and medication-related factors influence long-term overdose risk [45]. Well-structured opioid-risk stratification may help reduce opioid-related harm while preserving effective analgesia in cancer and palliative care settings [46]. For these patients, OFA should not be interpreted as mandatory opioid exclusion; rather, analgesia should be individualized through multimodal non-opioid strategies, appropriate rescue opioid availability, careful prescribing, and post-discharge monitoring [45,46]. Overall, safe patient selection for OFA and OSA requires validated risk-assessment tools, shared decision-making, procedure-specific planning, contraindication screening, rescue analgesia pathways, and post-discharge monitoring. Opioid minimization is safest and most clinically useful when individualized according to patient risk, surgical context, recovery goals, and available perioperative support rather than applied as a universal opioid-elimination protocol.

Potential harms of opioid-free protocols

OFA and OSA may reduce opioid-related adverse effects, but complete opioid avoidance can introduce clinically important risks when applied rigidly or without appropriate patient selection. The principal concern is inadequate analgesia, particularly after procedures associated with severe somatic, visceral, thoracic, orthopedic, spinal, or neuropathic pain [4,5]. Undertreated pain may increase sympathetic activation, hemodynamic instability, impaired ventilation, sleep disruption, delayed mobilization, patient dissatisfaction, and the risk of persistent postsurgical pain [14]. Therefore, opioid-free protocols should include clear rescue analgesia pathways and should not delay opioid administration when pain remains uncontrolled despite multimodal therapy [31].

Non-opioid agents used in OFA protocols also require careful selection and monitoring. Alpha-2 agonists may cause bradycardia, hypotension, sedation, or delayed recovery; ketamine may cause psychotomimetic effects, nausea, hypertension, or emergence reactions; intravenous lidocaine requires dose control because of neurologic and cardiac toxicity risk; nonsteroidal anti-inflammatory drugs and cyclooxygenase-2 inhibitors may be limited by renal, gastrointestinal, cardiovascular, or bleeding concerns; and gabapentinoids may increase sedation, dizziness, respiratory vulnerability, and delayed mobilization, particularly in older or frail patients [16,19]. Regional anesthesia and fascial plane blocks may reduce opioid exposure but can be associated with block failure, rebound pain, local anesthetic systemic toxicity, vascular puncture, infection, hematoma, nerve injury, pneumothorax, or delayed recognition of surgical complications [25,26].

OFA is not equally suitable for all patients or procedures. Patients with chronic opioid use, opioid tolerance, complex pain syndromes, major oncologic surgery, extensive orthopedic, thoracic, or spinal procedures, severe baseline pain, frailty, or limited postoperative support may require individualized opioid-sparing rather than strictly opioid-free plans [5,42]. Khan and Singh described a non-opioid medication combination for OFA in spine surgery and reported perioperative analgesia, hemodynamic stability, patient satisfaction, and postoperative recovery outcomes; however, this case-series/narrative evidence should be interpreted cautiously because it cannot establish comparative effectiveness or broad safety [37]. Thus, OFA should be implemented as a flexible component of multimodal and ERAS-based care, supported by contraindication screening, patient counseling, monitoring, reassessment of pain and function, and explicit rescue analgesia pathways.

Limitations and future directions

As a scoping review, this article maps the breadth, themes, and gaps in the available literature rather than estimating pooled intervention effects or assigning certainty-of-evidence ratings. The findings should therefore be interpreted as an evidence map and clinically oriented synthesis rather than as a definitive comparative-effectiveness assessment. The current evidence on OFA and OSA remains heterogeneous in study design, surgical population, drug combinations, dosing schedules, regional anesthesia techniques, rescue analgesia protocols, outcome definitions, and adverse-event reporting. This heterogeneity limits direct comparison across studies and prevents broad conclusions about a single optimal opioid-free or opioid-sparing protocol. Short-term outcomes, including pain scores, opioid consumption, postoperative nausea and vomiting, and length of stay, are reported more consistently than long-term outcomes such as persistent postsurgical pain, functional recovery, quality of life, readmission, and long-term opioid use.

Future studies should move beyond opioid consumption as the primary marker of success and prioritize recovery quality, functional outcomes, and patient safety. Adequately powered multicenter trials are needed using procedure-specific OFA and OSA protocols, standardized non-opioid dosing regimens, clearly defined rescue analgesia pathways, and consistent adverse-event reporting. Recovery-quality outcomes should include pain at rest and during movement, nausea, sedation, respiratory safety, bowel recovery, sleep quality, mobilization, oral intake, discharge readiness, patient satisfaction, readmission, persistent postsurgical pain, and post-discharge opioid use. Comparative studies should also evaluate strict OFA versus opioid-sparing balanced analgesia to clarify which patients benefit from complete opioid avoidance and which require limited opioid rescue as part of safer individualized care. Future OFA and OSA trials should treat validated recovery-quality measures, functional recovery, adverse events, and patient satisfaction as primary or co-primary outcomes, rather than relying primarily on postoperative opioid consumption.

## Conclusions

This scoping review shows that OFA and OSA represent a shift from opioid-centered pain control toward multimodal, recovery-focused perioperative care. The key points of this review are that opioid minimization may reduce opioid-related adverse effects, but its success depends on effective analgesia, patient safety, functional recovery, and appropriate patient selection. Systemic non-opioid agents, regional anesthesia, fascial plane blocks, and enhanced recovery pathways are most useful when combined within procedure-specific protocols. Complete opioid avoidance should not be the goal for every patient. Opioids should remain available when needed for rescue analgesia, severe pain, or hemodynamic stability. Future practice should emphasize individualized analgesia, standardized recovery outcomes, risk stratification, multidisciplinary planning, and structured post-discharge monitoring. Potential harms of OFA include undertreated pain, delayed rescue analgesia, adverse effects from non-opioid adjuncts, and complications related to regional anesthesia; therefore, recovery quality and individualized analgesic safety should be prioritized over opioid consumption alone. Overall, opioid-free and opioid-sparing pain control should be considered flexible clinical strategies rather than universal replacements for balanced anesthesia.
